# Prevalence and factors associated with preterm birth at kenyatta national hospital

**DOI:** 10.1186/s12884-018-1740-2

**Published:** 2018-04-19

**Authors:** Peter Wagura, Aggrey Wasunna, Ahmed Laving, Dalton Wamalwa, Paul Ng’ang’a

**Affiliations:** 1 Department of Paediatrics and Child Health, College of Health Sciences, University of Nairobi, P.O. Box 19676-00202, Nairobi, Kenya; 2 Division of Neglected Tropical Diseases, Ministry of Health, P.O. Box 20750-00202, Nairobi, Kenya

**Keywords:** Preterm birth, Prematurity, Preterm delivery

## Abstract

**Background:**

The World Health Organization estimates the prevalence of preterm birth to be 5–18% across 184 countries of the world. Statistics from countries with reliable data show that preterm birth is on the rise. About a third of neonatal deaths are directly attributed to prematurity and this has hindered the achievement of Millennium Development Goal-4 target. Locally, few studies have looked at the prevalence of preterm delivery and factors associated with it. This study determined the prevalence of preterm birth and the factors associated with preterm delivery at Kenyatta National Hospital in Nairobi, Kenya.

**Methods:**

A cross-sectional descriptive study was conducted at the maternity unit of Kenyatta National Hospital in Nairobi, Kenya in December 2013. A total of 322 mothers who met the eligibility criteria and their babies were enrolled into the study. Mothers were interviewed using a standard pretested questionnaire and additional data extracted from medical records. The mothers’ nutritional status was assessed using mid-upper arm circumference measured on the left. Gestational age was assessed clinically using the Finnstrom Score.

**Results:**

The prevalence of preterm birth was found to be 18.3%. Maternal age, parity, previous preterm birth, multiple gestation, pregnancy induced hypertension, antepartum hemorrhage, prolonged prelabor rupture of membranes and urinary tract infections were significantly associated with preterm birth (*p* = < 0.05) although maternal age less < 20 years appeared to be protective. Only pregnancy induced hypertension, antepartum hemorrhage and prolonged prelabor rupture of membranes remained significant after controlling for confounders. Marital status, level of education, smoking, alcohol use, antenatal clinic attendance, Human Immunodeficiency Virus status, anemia, maternal middle upper arm circumference and interpregnancy interval were not associated with preterm birth.

**Conclusions:**

The prevalence of preterm birth in Kenyatta National Hospital was 18.3%. Maternal age ≤ 20 years, parity > 4, twin gestation, maternal urinary tract infections, pregnancy induced hypertension, antepartum hemorrhage and prolonged prelabor rupture of membranes were significantly associated with preterm birth. The latter 3 were independent determinants of preterm birth. At-risk mothers should receive intensified antenatal care to mitigate preterm birth.

## Background

Of the estimated 130 million babies born each year globally, approximately 15 million are born preterm. Prematurity is a major cause of neonatal mortality and morbidity as well as a significant contributor to long term adverse health outcomes. Prematurity is a major hindrance to the attainment of the Millennium Development Goals (MDG)-4 target given its high contribution to neonatal mortality. The survival chances of babies born preterm vary significantly depending on where they are born. The risk of neonatal death due to complications of preterm birth is at least 12 times higher for an African baby than for a European baby. Preterm birth (PTB) is a global problem with prevalence ranging between 5 and 18% across 184 countries. The highest rates of preterm birth are in Sub-Saharan Africa and Asia which account for half the world’s births, more than 60% of the world’s preterm babies and over 80% of the world’s 1.1 million neonatal deaths annually due to complications related to preterm birth. Though most countries especially the low and middle income ones lack reliable data on preterm birth, nearly all of those with reliable trend data show an increase in preterm birth rates over the past 20 years. Indeed, all but 3 out of 65 countries in the world with reliable trend show an increase in preterm birth rates in the last 20 years. Significant progress has been made in the care of premature infants but not in reducing the prevalence of preterm birth which is generally on the rise. Causes of preterm birth are unknown in over 50% of spontaneous preterm labor while mechanisms of preterm labor remain poorly understood [1–7]. Identifying and understanding the risk factors for preterm birth has the potential to help address this problem.

Kenya like most developing countries lacks reliable data on the burden of preterm delivery. Kenyatta National Hospital (KNH) is the largest regional referral and handles many high risk pregnancies some of which result in preterm birth. Despite this, few published studies on the burden of preterm birth and the factors associated with it exist locally. This study aimed to determine the prevalence of preterm birth and the factors associated with PTB. The findings of the study are presented in this article.

## Methods

### Study design

A hospital based descriptive cross-sectional study was conducted using interviewer administered questionnaire. Additional information was obtained from medical records of the mothers and babies.

### Study area

KNH is the largest referral hospital in Kenya and Eastern and Central Africa and also serves as a teaching hospital for the University of Nairobi and the Kenya Medical Training College. It is located in Nairobi which is the capital city of Kenya with a population of about 4 million. The hospital has a busy maternity unit registering over 10,000 deliveries annually. It also has a busy newborn unit (NBU) which offers specialised neonatal care. Being a teaching and referral hospital, KNH handles many high risk pregnancies whose outcomes often include preterm birth.

### Participants

The study population comprised of all mothers who had live births at Kenyatta National Hospital and their newborns. A total of 322 mothers who met the eligibility criteria were enrolled into the study. These mothers delivered a total of 331 babies 18 of which were twins.

### Data collection

All mothers who had live births at KNH in December 2013 were identified using the birth register within 24 h of delivery. Systematic sampling was used to recruit mother-baby pairs. Mothers were traced to the postnatal wards. Informed consent was obtained from the mothers and babies admitted to the newborn unit were also traced. A standard pretested questionnaire was administered to the mothers while additional data was obtained from the mothers’ and babies’ medical records as required. The records examined for additional data included the mothers’ antenatal and admission records and the babies’ medical records for those admitted in the NBU after delivery. Information collected from the mother included maternal age, marital status, level of education, occupation, smoking and alcohol use during pregnancy, parity, date of last normal menstrual period, date of current and preceding delivery (for calculation of interpregnancy interval) and history of previous preterm birth. Information obtained from medical records included antenatal clinic (ANC) attendance and number of visits, Human Immune Deficiency (HIV) status, hemoglobin level, mode of delivery, onset of labor (spontaneous or medically indicated), pregnancy outcome (singleton or multiple), birthweight (to nearest 10 g), baby’s gender, prelabor rupture of membranes (PROM) for > 18 h, pregnancy induced hypertension (PIH), antepartum hemorrhage (APH), history of burning sensation during pregnancy or treatment for urinary tract infection (UTI). Anemia was defined as hemoglobin level of < 10 g/dl. PIH was defined clinically as a blood pressure of > 140/90 mmHg after 20 weeks of gestation with or without proteinuria and/or edema as diagnosed and documented by the attending clinician. APH was defined as any vaginal bleeding in the mother after 24 weeks of gestation as documented in the records by the attending clinician. UTI was defined as a documented clinical/laboratory diagnosis of UTI any time during the pregnancy and/or a positive history of treatment of burning sensation with micturition as reported by the mother. Maternal nutritional status was assessed by measuring the left mid-upper arm circumference (MUAC) using non-stretchable World Food Program MUAC tapes used for screening pregnant mothers. A low MUAC was defined as a measurement of less than 24 cm. Gestational age was calculated using a standard obstetric wheel based on menstrual dates and confirmed within 24 h of birth by clinical assessment using the Finnstrom Score. This method was developed by Finnstrom et al. in 1977. Seven (7) physical parameters which are scalp hair, skin opacity, length of fingernails, breast size, nipple formation, ear cartilage and plantar skin creases were used. This tool is not only easy to use but is also sensitive with an accuracy of +/− 2 weeks when administered within 24 h of birth [8, 9]. To limit observer bias, gestational assessment of all babies was done by only one research assistant trained by the principal investigator and aided by a printed pictorial scoring chart. For uniformity, gestational age used for analysis was based on Finnstrom score and not on menstrual dates. Preterm birth was defined as a gestation of less than 37 completed weeks. Prematurity was further categorized as extreme (less than 28 weeks), severe (28–31 weeks), moderate (32–33 weeks) and late preterm or near term (34–36 weeks).

### Data analysis

Data was entered into Microsoft Access database, cleaned and stored in a password protected external storage device. Data was analyzed using Stata 11.0. Mean, median, frequencies and percentages were reported to describe the variables and inferential statistics were used to establish associations between prematurity and the various risk factors using a chi-square analysis. Multivariate logistic regression was used to determine the factors independently associated with preterm birth.

## Results

### Background characteristics of participants

The mean maternal age was 26 ± 5 years with majority (89%) being aged 20 years and above. Most of the mothers (83%) were married. About 85% of the mothers had attained post-primary level of education. About 97% of the enrolled mothers had singleton deliveries while 82% delivered at term. Fifty three percent of all the babies in the study were males. The mean birth weight of term babies was 3059 ± 538 g. The median weight of preterm babies was 2110 g (IQR 1650–2400). The mean gestation was 39 ± 3 weeks and 33 ± 3 weeks for term and preterm babies respectively. Of the preterm births, 62% were late preterms (34–36 weeks), 19% were moderate preterms (32-33 weeks), 16% were severe preterm (28–31 weeks) and 3% were extreme preterm (< 28 weeks).

### Prevalence of preterm birth

The prevalence of preterm birth among live births was 18.3% (95% Confidence Interval (CI) of 14.1–22.5%).

### Socio-demographic characteristics

About 80% of mothers in the term and 90% in the preterm group were aged 20–34 years. Thirteen percent of mothers aged less than 20 years delivered at term compared to 3.4% who had preterm delivery and this was significant (*p* = 0.034). The proportions of mothers aged 35 years and above were similar in the two groups. There was no difference between the preterm and term groups in terms of marital status (*p* = 0.133), maternal level of education (*p* = 0.330), occupation (*p* = 0.823), smoking (*p* = 0.728), antenatal alcohol use (*p* = 0.501) and maternal MUAC (*p* = 0.651). None of the socio-demographic factors was significantly associated with preterm birth except maternal age less than 20 years which was negatively associated with preterm delivery (OR 0.236). Table 1 shows the relationship between the socio-demographic characteristics and preterm delivery.

**Table 1:** Socio-demographic characteristics

Factors	Term (*n* = 263) (%)	Preterm (*n* = 59) (%)	OR (95% CI)	*P*-value
Maternal age (years)				
< 20	34 (13.0)	2 (3.4)	0.236 (0.054–1.001)	0.034
20–34	210 (79.8)	53 (89.8)	Ref	
≥ 35	19 (7.2)	4 (6.8)	0.834 (0.272–2.555)	0.751
Marital status				
Unmarried	48 (18.3)	6 (10.2)	0.507 (0.206–1.248)	0.133
Married	215 (81.7)	53 (89.8)		
Level of education				
No formal/Primary	36 (13.7)	11 (18.6)	1.445 (0.687–3.039)	0.330
Post-primary	227 (86.3)	48 (81.4)		
Maternal occupation				
Unemployed	169 (64.3)	37 (62.7)	0.935 (0.521–1.679)	0.823
Employed/business	94 (35.7)	22 (37.3)		
Smoking during pregnancy				
Yes	3 (1.1)	1 (1.7)	1.494 (0.153–14.623)	0.728
No	260 (98.9)	58 (98.3)		
Alcohol in pregnancy				
Yes	16 (6.1)	5 (8.5)	1.429 (0.502–4.070)	0.501
No	247 (93.9)	54 (91.5)		
MUAC (cm)				
< 24	10 (3.8)	3 (5.1)	1.391 (0.369–5.252)	0.651
≥ 24	253 (96.2)	56 (94.9)		

### Previous pregnancy characteristics

Most mothers had a parity of less than four. Women with a parity of 4 or more were nearly 5 times more likely to deliver preterm compared to those whose parity was < 4 (*p* = 0.019; OR 4.709). About 35% of mothers who delivered before term had a history of previous preterm delivery compared to 16% of those who delivered at term and this was significant (*p* = 0.010). Approximately 6% of mothers in the preterm group and 11% in the term group had an interpregnancy interval of < 24 months but this was not statistically significant (*p* = 0.357)). The relationship between previous pregnancy characteristics and preterm birth is summarized in Table 2.

**Table 2:** Previous pregnancy characteristics

Factors	Term (*n* = 263) (%)	Preterm (*n* = 59) (%)	OR (95% CI)	*P*-value
Parity				
≥ 4	4 (1.5)	4 (6.8)	4.709 (1.143–19.407)	0.019
< 4	259 (98.5)	55 (93.2)		
Previous preterm				
Yes	20 (15.6)	12 (35.3)	2.945 (1.259–6.891)	0.010
No	108 (84.4)	22 (64.7)		
Interpregnancy interval (months)				
< 24	14 (10.9)	2 (5.7)	0.506 (0.110–2.342)	0.357
≥ 24	114 (89.1)	33 (94.3)		

### Antenatal factors

The proportion of mothers who did not attend ANC in the term and preterm groups was 2.3 and 3.4% respectively and this was not significant (*p* = 0.621). Mothers who had not had any antenatal care were one and a half times more likely to deliver preterm (OR 1.503). About 29% of mothers in term and 37% in preterm group had less than 3 antenatal visits but this was statistically insignificant (*p* = 0.256). Approximately 13% of preterm mothers and 12% of term mothers were HIV positive. There was no association between HIV status and preterm delivery (*p* = 0.834). The proportion of women who had anemia during pregnancy was the same for the two groups (*p* = 0886). Table 3 shows the relationship between the antenatal characteristics and preterm delivery.

**Table 3:** Antenatal characteristics

Factors	Term n (%)	Preterm n (%)	OR (95% CI)	*P*-value
ANC attendance				
Yes	257 (97.7)	57 (96.6)	1.503 (0.296–7.639)	0.621
No	6 (2.3)	2 (3.4)		
No. of ANC visits				
< 3	75 (29.2)	21 (36.8)	1.416 (0.776–2.584)	0.256
≥ 3	182 (70.8)	36 (63.2)		
HIV status				
Seropositive	29 (11.5)	7 (12.5)	1.099 (0.455–2.652)	0.834
Seronegative	223 (88.5)	49 (87.5)		
Hemoglobin (g/dl)				
< 10	65 (29.0)	14 (28.0)	0.951 (0.481–1.880)	0.886
≥ 10	159 (71.0)	36 (72.0)		

### Delivery factors

Approximately 40% of preterm deliveries were via Caesarean section (C/S) compared to 26% among those who delivered vaginally. Women who delivered via Caesarean section were nearly two times (OR 1.832) more likely to deliver preterm than those who delivered vaginally. Delivery via Caesarean section had significant but marginal association with preterm birth (*p* = 0.049). About 28 and 36% of mothers in the term and preterm group respectively had induced labor or medically indicated C/S. However, there was no association between onset of labour and preterm birth (*p* = 0.231). The proportion of twin pregnancy among women who delivered at term and preterm was 2 and 7% respectively and this was significant (*p* = 0.040). Twin pregnancy conferred nearly a 4-fold increase in the risk of preterm birth (OR 3.753). Table 4 shows the relationship between the delivery characteristics and preterm delivery.

**Table 4:** Delivery characteristics

Factors	Term n (%)	Preterm n (%)	OR (95% CI)	*P*-value
Mode of delivery				
C/S	68 (25.9)	23 (39.0)	1.832 (1.014–3.310)	0.049
Vaginal	195 (74.1)	36 (61.0)		
Onset of labour				
Induced/Medical C/S	73 (27.8)	21 (35.6)	1.438 (0.791–2.614)	0.231
Spontaneous	190 (72.2)	38 (64.4)		
Pregnancy outcome				
Twins	5 (1.9)	4 (6.8)	3.753 (1.016–14.427)	0.040
Singleton	258 (98.1)	55 (93.2)		

### Obstetric factors

About 32 and 8% of mothers in the preterm and term groups had PIH while 13 and 5% of mothers in the two groups had APH respectively. Mothers with PIH and those with APH had a 5-fold and 3-fold increase in risk of preterm birth (OR 5.203 and 2.790). Approximately 27% of mothers who had preterm delivery and 8% of those who delivered at term had a history of PROM for more than 18 h while 47.5% of mothers in preterm group and 32% of those in the term group respectively reported having had UTI or burning sensation with micturition during pregnancy. As shown in Table 5, all these factors were significantly associated with preterm birth (*p* < 0.05).

**Table 5:** Obstetric characteristics

Factors	Term (*n* = 263) (%)	Preterm (*n* = 59) (%)	OR (95% CI)	*P*-value
Pre-eclampsia				
Yes	22 (8.4)	19 (32.2)	5.203 (2.586–10.4690)	< 0.001
No	241 (91.6)	40 (67.8)		
APH				
Yes	14 (5.3)	8 (13.6)	2.790 (1.112–6.997)	0.023
No	249 (94.7)	51 (86.4)		
PROM>18Hrs				
Yes	22 (8.4)	16 (27.1)	4.059 (1.974–8.349)	< 0.001
No	240 (91.6)	43 (72.9)		
History of UTI				
Yes	84 (31.9)	28 (47.5)	1.925 (1.085–3.414)	0.024
No	179 (68.1)	31 (52.5)		

### Independent determinants of preterm birth

Maternal age, parity, previous preterm birth, twin gestation, UTI, PIH, prolonged PROM and APH were found to be significantly associated with preterm birth. However, on multivariate logistic regression only PIH, APH and prolonged PROM remained significant. The risk of preterm birth increased 8-fold with PIH (OR 7.805), 5-fold if the mother had prolonged PROM (OR 5.319) and 4-fold with APH (OR 4.264) after controlling for confounders. The multivariate logistic regression is summarized in Table 6.

**Table 6:** Multivariate logistic regression of significant factors

Variables	AOR (95% Confidence Interval)	*P* value
Maternal age < 20 years	0.183 (0.032–1.055	0.057
Parity	0.716 (0.118–4.336)	0.716
Twin gestation	1.908 (0.482–7.552)	0.358
UTI	1.775 (0.657–4.795)	0.258
Prolonged PROM	5.319 (2.320–12.195)	< 0.001
Pregnancy induced hypertension	7.805 (3.686–16.525)	< 0.001
APH	4.264 (1.517–11.986)	< 0.001
Previous preterm birth	1.407 (0.721–2.746)	0.317

## Discussion

Most developing countries lack reliable data on the prevalence of preterm birth [2, 4]. This study aimed to determine the prevalence of preterm birth and associated factors at the largest teaching and referral hospital in Nairobi, Kenya. Our findings demonstrate that preterm birth is a significant health problem in this population with a hospital based prevalence rate of 183 per 1000 live births and that PIH, APH and prolonged PROM are independently associated with PTB. The high rate of preterm birth in this study is in agreement with World Health Organization (WHO) estimates that show that the highest rates are in sub Saharan Africa and South Asia and similar to the finding of other studies in India, Zimbabwe and Malawi [2, 10–12]. However, this PTB rate is higher than would be expected for community based studies. Compared to low and medium level health facilities in which most normal and uncomplicated deliveries are conducted, KNH being a major referral hospital handles more complicated deliveries, a significant proportion of which are preterm. Consequently, when estimating the PTB rate, the numerator is higher in relation to the denominator for the tertiary hospital resulting in a higher prevalence. The prevalence of preterm birth in the current study is much higher than that reported by Olugbenga and others in a study in a teaching hospital in Nigeria [13]. The difference in PTB rates between our study and the study done by Olugbenga et al. in almost similar setting in the sense of both being teaching hospitals could be explained by the distinct approaches in estimating the gestational age of the babies. While their study excluded mothers who were unsure of dates, those who had a discrepancy of more than 2 weeks between gestation by dates and Ballard’s assessment as well as those who had multiple gestation, our study relied wholly on the clinical gestational age assessment based on Finnstrom score. It is likely that our approach overestimated the prevalence of PTB while that of Olugbenga et al. may have underestimated the same.

The current study did not show any association between the maternal socio-demographic factors except maternal age < 20 years that appeared to be marginally protective. Though our findings showed a marginal negative association between maternal age < 20 years and PTB (*p* value =0.034, OR = 0.236), this is both unexpected and different from other studies [11–14]. Although about 11% of all mothers were aged < 20 years, less than 1% had preterm birth. The number of women who delivered prematurely in this regard was too small to authoritatively detect significant association with preterm birth and may have inadvertently resulted in the negative association in our study. Previous preterm delivery was associated with preterm birth and this was similar to the findings of other studies [13, 14]. Though the exact mechanism for this is not well established, it may be due to persistence of unidentified factors such as subclinical infections as well as underlying disorders such as hypertension, obesity or diabetes in some women precipitating preterm delivery [1, 15]. The current study demonstrated that mothers with a parity of ≥4 were 4 times more likely to deliver prematurely. This finding is similar to that of previous studies which had shown that multiparaous women were more likely to deliver preterm [13, 14]. High parity is likely to increase the risk of preterm delivery due to uterine changes such as myometrial stretching from previous pregnancies. Some of the mothers with high parity may also have had a bad obstetric history which may be due to unidentified factors that may persist in subsequent pregnancies. Interpregnancy interval had no association with preterm birth. This was different from the findings of Gordon and colleagues and Agustin Conde and others but similar to that of J Etuk and others in Nigeria [14, 16, 17]. It is possible that women in our setting recover faster from the effect of previous pregnancy and this may be due to intensified nutritional care of mothers soon after delivery which is a common practice locally.

Delivery via Caesarean Section was significantly associated with preterm birth but onset of labor was not. This was similar to the finding of Olugbenga et al. [13]. Operative delivery has no causal relationship with preterm birth but rather is as a result of indicated delivery for maternal or fetal reasons occasioned by obstetric complications such as PIH and APH as observed in this study.

Twin gestation was significantly associated with preterm birth in this study. This is similar to the findings of J Etuk and others [14]. Mutiple gestation is associated with uterine overdistension and this may result in spontaneous preterm labour. In addition other complications such as pre-eclampsia and polyhydramnios are more likely to occur with multiple gestations and thus contribute to iatrogenic preterm birth [1].

ANC attendance as well as number of antenatal visits was not associated with preterm birth in our study. This is different from what Feresu A et al. had reported in Zimbabwe [11]. This may have been due to the Focused Antenatal Care (FANC) approach in Kenya which has emphasized the need to have four targeted antenatal visits which ensures women start ANC attendance much earlier [18]. Maternal HIV status was not associated with preterm delivery in the current study. This finding was similar to that of J Coley and colleagues in Tanzania and J Ndirangu and others in South Africa [19, 20]. It is possible that with increasing availability and use of antiretroviral drugs for prophylaxis and treatment of HIV in pregnancy, the impact of HIV on pregnancy outcomes including risk of preterm birth may have been reduced. Anemia in pregnancy had been associated with preterm birth in some studies but not in others [13, 14, 21]. Our study did not show any association between preterm birth and anemia. With the FANC approach, all pregnant mothers receive iron and folate supplements as early as possible and this reduces the risk of complications related to anemia including preterm birth. A low maternal MUAC was not associated with preterm birth. This finding was different from that of Sebayang et al. in Indonesia and Kalanda et al. in Malawi [21, 22]. One possible reason for this difference is that most women in the current study were from an urban setting compared with the rural setting of the other two studies. UTI in pregnancy was associated with premature birth. This was similar to the findings of studies in Iran and Nigeria [9, 15]. Due to morphological and functional changes that occur in pregnancy, stasis of urine favors UTI. Like other infections, UTI stimulate production of cytokines which may induce preterm labor through release of prostaglandins.

Results of the current study demonstrated that after controlling for confounders, prolonged PROM, PIH and APH remained significantly associated with preterm birth. These findings are similar to those reported in other studies. PROM has been associated with chorioamnionitis which may be subclinical and chlamydial vaginitis. Microorganisms that cause bacterial vaginosis can easily ascend in prolonged PROM and cause intrauterine infections. It is postulated that subclinical chorioamnionitis and other unidentified infections may trigger the release of inflammatory mediators such as interleukin 1 leading to release of prostaglandins from the uterine decidua that ultimately induce preterm labor. PIH which is one of the major obstetric complications was significantly associated with PTB in the current study. Though the pathophysiology of this condition remains poorly understood, uteroplacental ischemia is a plausible explanation for the poor pregnancy outcomes associated with PIH including preterm delivery and low birthweight. Furthermore, PIH is a common reason for indicated preterm deliveries and this may explain its association with PTB even though this may not be causal in nature. Like PIH, APH is also a major contributor to indicated preterm deliveries whether vaginally or operatively without necessarily having a temporal relationship with PTB [1, 9, 15]. This study identifies mothers with prolonged PROM, PIH and APH as a high risk group for PTB. These are largely modifiable factors and should form a good basis for prenatal interventions and better management geared towards reducing the burden of PTB.

### Limitations of the study

Only mothers who had live births were interviewed and their babies assessed for gestational age. The study did not address factors associated with preterm stillbirth. UTI in pregnancy was partly based on mothers’ self report of symptoms and not on laboratory confirmation and therefore over-reporting was likely. Clinical assessment of gestation using the Finnstrom method that solely relied on physical characteristics is also a limitation of this study. Use of secondary data for some variables is another limitation of our study.

## Conclusions

Preterm birth among women delivering at KNH in Nairobi Kenya is a significant problem. Prolonged PROM, PIH and APH are independent determinants of preterm birth. Better management of these obstetric complications and research to elucidate the mechanisms by which they cause preterm birth, offers a practical approach of reducing the high preterm birth rates.

## Abbreviations

ANC: Antenatal clinic
AOR: Adjusted odds ratio
APH: Antepartum hemorrhage
C/S: Caesarean section
CI: Confidence interval
DOMC: Division of Malaria Control
DRH: Division of Reproductive Health
FANC: Focused antenatal care
HIV: Human immunodeficiency virus
JHPIEGO: John Hopkins Program for International Education in Gynecology and Obstetrics
KNH: Kenyatta national hospital
MDG: Millennium development goal
MOH: Ministry of Health
MUAC: Mid upper arm circumference
NBU: Newborn unit
OR: Odds ratio
PIH: Pregnancy induced hypertension
PROM: Prelabor rupture of membranes
PTB: Preterm Birth
SVD: Spontaneous vertex delivery
UTI: Urinary tract infection
WHO: World Health Organization

## References

[1] Goldenberg RL, Culhane JF, Iams JD, Romero R (2008). Epidemiology and causes of preterm birth. Lancet.

[2] March of Dimes/WHO (2012). Born too soon-the global action report on preterm birth.

[3] Lawn JE, Cousens S, Zupan J (2008). 4 million neonatal deaths: when? Where? Why?. Lancet.

[4] Blencowe H, Cousens S, Oestergaard M (2012). National, regional and worldwide estimates of preterm birth rates in the year 2010 with time trends for selected countries since 1990: a systematic analysis. Lancet.

[5] United Nations General Assembly (2000). United Nations millennium declaration.

[6] Martines J, Paul VK, Bhutta ZA, Koblinsky M, Soucat A, Walker N, Bahl R, Fogstad H, Costello A (2005). Neonatal survival: a call for action. Lancet.

[7] Lawn JE, Kerber K, Enweronu-Laryea C, Bateman O (2009). Newborn survival in low resource settings--are we delivering?. BJOG.

[8] Finnström O (1977). Studies on maturity in newborn infants. ActaPaediatrScand.

[9] Goyal SC, Tak SK, Bhandari B (1989). Determination of gestational age: comparative accuracy of different methods. Indian J Pediatr.

[10] Shubhada A, Kambale SV, Phalke BD (2013). Determinants of preterm labour in a rural medical college hospital in western Maharashtra. NJOG.

[11] Feresu SA, Harlow SD, Welch K, Gillespie RW (2004). Incidence of and socio-demographic risk factors for stillbirth, preterm birth and low birthweight among Zimbabwean women. Paediatr Perinat Epidemiol.

[12] van den Broek NR, Jean-Baptiste R, Neilson JP (2014). Factors associated with preterm, early preterm and late preterm birth in Malawi. PLoS One.

[13] Olugbenga A, Mokuolu BM, Suleiman OO, Adesiyun A, Adeniyi B (2010). Prevalence and determinants of pre-term deliveries in the University of Ilorin Teaching Hospital, Ilorin, Nigeria. Pediatric Report.

[14] Etuk SJ, Etuk IS, Oyo-Ita AE (2005). Factors influencing the incidence of preterm birth in Calabar, Nigeria. Niger J Physiol Sci.

[15] Muglia LJ, Katz M (2010). The enigma of spontaneous preterm birth. N Engl J Med.

[16] Smith GCS, Pell JP, Dobbie R (2003). Interpregnancy interval and risk of preterm birth and neonatal death: retrospective cohort study. BMJ.

[17] Conde-Agudelo A, Rosas-Bermãdez A, Kafury-Goeta A (2006). Birth spacing and risk of adverse perinatal outcomes. JAMA.

[18] MOH-DRH, DOMC, JHPIEGO (2002). Focused antenatal care and malaria in pregnancy: orientation package.

[19] Coley JL, Kapiga S, Hunter D (2001). The association between maternal HIV-1 infection and pregnancy outcomes in Dar es Salaam, Tanzania. BJOG.

[20] Ndirangu J, Newell M-L, Bland RM, Thorne C (2012). Maternal HIV infection associated with small-for-gestational age infants but not preterm births: evidence from rural South Africa. Hum Reprod.

[21] Sebayang S, Dibley M, Kelly P, Shanka A, Anuraj H (2012). Determinants of low birth weight, and small-for-gestational-age and preterm birth in Lombok, Indonesia: analyses of the birth weight cohort of the SUMMIT trial. Trop Med Int Health.

[22] Kalanda BF (2007). Maternal anthropometry and weight gain as risk factors for poor pregnancy outcomes in a rural area of southern Malawi. Malawi Med J.

